# No apparent transmission of transgenic α–synuclein into nigrostriatal dopaminergic neurons in multiple mouse models

**DOI:** 10.1186/s40035-015-0046-9

**Published:** 2015-12-03

**Authors:** Namratha Sastry, Wang Zheng, Guoxiang Liu, Helen Wang, Xi Chen, Michael Cai, Parth Contractor, Carmelo Sgobio, Lixin Sun, Chengsong Xie, Huaibin Cai

**Affiliations:** Transgenics Section, Laboratory of Neurogenetics, National Institute on Aging, National Institutes of Health, Building 35, Room 1A112, MSC 3707, 35 Convent Drive, Bethesda, MD 20892-3707 USA; Unit on Synapse Development Plasticity, Clinical Brain Disorder Branch, National Institute of Mental Health, National Institutes of Health, Bethesda, MD 20892 USA; Present addresses: Feinberg School of Medicine, Northwestern University, Chicago, IL 60611 USA; Present addresses: Swarthmore College, Swarthmore, PA 19081 USA; Present addresses: Centennial High School, Elicott City, MD 21042 USA; Present addresses: George Washington University, Washington, DC 20052 USA

**Keywords:** Parkinson’s disease, α-synuclein, Propagation, Dopaminergic neurons, Transgenic mice

## Abstract

**Background:**

α–synuclein (α–syn) is the main component of intracytoplasmic inclusions deposited in the brains of patients with Parkinson’s disease (PD) and certain other neurodegenerative disorders. Recent studies have explored the ability of α–syn to propagate between or across neighboring neurons and supposedly “infect” them with a prion–like mechanism. However, much of this research has used stereotaxic injections of heterologous α–syn fibrils to induce the spreading of inclusions in the rodent brains. Whether α–syn is able to transmit from the host cells to their neighboring cells in vivo is unclear.

**Methods:**

Using immunestaining, we examined the potential propagation of α–syn into nigrostriatal dopaminergic (DA) neurons in three lines of transgenic mice that overexpress human wild–type α–syn (hα–syn) in different neuron populations.

**Results:**

After testing for three different routes by which hα–syn propagation might occur, we were unable to find any evidence that hα–syn behaved like a prion and could be transmitted overtime into the DA neurons initially lack of hα–syn expression.

**Conclusions:**

In transgenic mice hα–syn does not have the ability to propagate at pathologically significant levels between or across neurons. It must be noted that these observations do not disprove the studies that show its prion–like qualities, but rather that propagation is not detectable in transgenic models that do not use any injections of heterologous proteins or viral vectors to induce a spreading state.

## Background

Parkinson’s disease (PD) is the second most common neurodegenerative disease, causing debilitating motor and non–motor symptoms [1, 2]. PD is pathologically characterized by the death of nigrostriatal dopaminergic neurons in the ventral *substantia nigra pars compacta* (SNc) of the midbrain, as well as the presence of intracytoplasmic inclusions known as Lewy bodies (LBs) and Lewy neurites (LNs). The main component of these inclusions is α–synuclein (α–syn) [3]. α–syn is a small 140 amino acid protein that is thought to play a role in synaptic vesicle release [4]. Both missense and multiplication mutations of α–syn are linked to early onset familial forms of PD [5]. How mutant α–syn leads to SNc DA neuron loss and LB/LN formation has been under intense investigation ever since.

PD patient brains seem to show a stereotypical appearance of LB/LN pathology that can be mapped into various stages of disease evolution: lesions first appear in the glossopharyngeal and vagal nerves, continue to the SNc DA neurons, and eventually cover the primary sensory and motor cortices [6]. Subsequent studies have hypothesized that α–syn intercellular propagation may be responsible for this stereotypical pathology and have provided evidence both in vitro and in vivo [7–10]. The in vivo studies have primarily focused on using intracerebral inoculations of diseased brain homogenate or preformed α–syn fibrils to study development and progression of PD pathology [9, 10]. In wild–type control mice, injections of preformed fibrils of α–syn are sufficient to initiate LB/LN pathology in regions anatomically connected to the site of injection [9]. Such intracerebral injections can also accelerate the formation of LBs and LNs in otherwise asymptotic mice [10] and can be seen over serial passages of inoculations [11]. This has been further confirmed by a study where inoculation with homogenate from either A53T human α–syn transgenic mouse brains or multiple system atrophy patient brains resulted in disease pathology in mice that do not otherwise develop any spontaneous illness [12]. Moreover, this phenomenon has been studied in primates, and propagation is evident in macaque monkeys in addition to rodents [13]. Increasing evidence thus suggests that α–syn potentially behaves in a prion–like manner, where mutated α–syn can be transmitted from cell to cell and spread the pathology [14].

While these studies provide very compelling results to show the prion–like qualities of α–syn, much of the methodology involves artificial injections and inoculations. This triggered us to determine if α–syn propagation could be observed in mice that overexpress human wild–type α–syn (hα–syn), without the need for any injections. Therefore, we generated multiple lines of transgenic mice that overexpress hα–syn in different neuron populations inside and outside of the SNc. We then examined three different routes by which α–syn may propagate into the SNc DA neurons, including long–range propagation from anatomically separated regions, short–distance transmission from presynaptic spiny projection neurons (SPNs), and neighboring DA neurons. Unlike previous inoculation experiments, we found no evidence that α–syn could propagate and possess prion–like qualities in any of our three modes of study, thus questioning α–syn propagation as the method of disease progression in PD.

## Methods

### Ethics statement

This study was carried out in strict accordance with the recommendations in the Guide for the Care and Use of Laboratory Animals of the National Institutes of Health. The protocol was approved by the Institutional Animal Care and Use Committees of the National Institute of Child Health and Human Development, NIH (Permit Number: 13–040). All surgery was performed under ketamine anesthesia, and all efforts were made to minimize suffering.

### Animals

To generate *tetO–SNCA* transgenic mice, human wild–type α-syn (*SNCA*) cDNA coding region was inserted into the mouse prion protein (pPrP)–tetO gene expression vector (a gift from Dr. David Borchelt, University of Florida, Gainesville, FL), which is controlled by the tetracycline-responsive promoter (tetP) [15]. The tetO-SNCA expression construct was then purified and microinjected into fertilized oocytes derived from C57BL/6 J mice. The founder mice were crossed with wild-type C57BL/6 J mice to produce the F1 generation. *Pitx3–tTA* knock–in mice were created as described previously [16]. *Drd1a–rtTA* mice were obtained from Jackson Laboratories (Bar Harbor, ME). All mice were housed in a 12 h light/dark cycle and fed regular diet *ad libitum*. All mouse work follows the guidelines approved by the Institutional Animal Care and Use Committees of the National Institute of Child Health and Human Development, NIH.

### Genotyping

Genomic DNA was prepared from tail biopsy using DirectPCR Lysis Reagent (Viagen Biotech, Inc., Los Angeles, CA) and subjected to PCR amplification using specific sets of PCR primers for each genotype, including *Pitx3–tTA* transgenic mice (*Pitx3*–F: GACTGGCTTGCCCTCGTCCCA and *Pitx3*–R: GTGCACCGAGGCCCCAGATCA), *tetO–SNCA* transgenic mice (PrpEx2–F: TACTGCTCCATTTTGCGTGA and SNCA–R: TCCAGAATTCCTTCCTGTGG), *Drd1a–rtTA* transgenic mice (14915–F: ACCGGAAGTGCTTTCCTTCT and 14916–R: CGACTTGATGCTCTTGATCTTCC).

### Immunohistochemistry and light microscopy

Mice were sacrificed and then perfused via cardiac infusion with 4 % paraformaldehyde in cold PBS, followed by post–fixation in the same solution overnight. To obtain sections, brain tissues were removed and submerged in 30 % sucrose for 24 h and sectioned at 30 μm thickness using a cryostat (Leica CM1950, Buffalo Grove, IL). Antibodies specific to TH (rabbit polyclonal, 1:1000, Pel–Freez, Rogers, AR), human α–synuclein (mouse monoclonal, syn211, 1:500, Santa Cruz Biotech, Santa Cruz, CA) were used as suggested by manufacturers. Alexa 488 and Alexa 546–conjugated secondary antibodies (1:500, Life Technologies, Grand Island, NY) were used to visualize the staining. Fluorescence images were captured using a laser scanning confocal microscope (LSM 510; Zeiss, Thornwood, NJ). The images were presented as either a single optic layer after acquisition in z–series stack scans at 2–3 μm intervals from individual fields or displayed as maximum intensity projections to represent confocal stacks.

### Image analysis

For the quantitative co–localization assessments, images from serial sections were taken and exported to ImageJ (NIH, Bethesda, MD) for imaging analyses. Each image was split into individual channels for SNCA (488 nm) and TH (546 nm). Cell bodies positive for TH were first selected using the polygon selection tool and then subjected to measurement by mean optical intensities. The mean intensities were then compared to the identical regions in SNCA channel. SNCA intensities below a set threshold were counted as being negative. The overall percentages of positive SNCA cells were then compared between the ages of 1 m and 16–18 m.

### Stereology

According to the mouse brain in stereotaxic coordinates (3rd edition, Keith B.J. Franklin and George Paxinos), a series of coronal sections across the midbrain (30 μm per section, every fourth section from Bregma −2.54 mm to −4.24 mm) were chosen and processed for TH staining as described above and visualized using a widefield microscope (Axio Imager A1; Zeiss). We examined 16 sections per brain at 40x magnification. The number of TH positive neurons was assessed using the Optical Fractionator Workflow in Stereo Investigator 11 (MicroBrightField Inc, Williston, VT). The sampling scheme was designed to have coefficient of error (CE) less than 10 % in order to get reliable results. A pilot counting of samples was performed to achieve a total marking of >200 cells, which generally yields CE <10 %. Once the pilot cells counting had completed, the CE was calculated. The counting parameters would be adjusted based on the CE value. To achieve CE <10 %, normally 12 series sections with total 100 counting frames and on average 2 cells per frame would be counted. The final parameters for these studies were: grid size 150x120μm and frame size 50x50μm. Three or more mice were used per genotype at each time point.

### Tissue fractionation and Western blot

Striatum tissues were homogenized with 10 volumes of sucrose buffer (0.32 M sucrose, 1 mM NaHCO3, 1 mM MgCl2, and 0.5 mM CaCl2, plus protease and phosphatase inhibitor cocktails) and centrifuged at 10,000 g for 10 min. Protein concentrations in supernatant were measured by BCA (Thermo Fisher Scientific). Proteins were size–fractioned by 4–12 % NuPage BisTris– polyacrylamide gel electrophoresis (Life Technologies) using MES running buffer (Life Technologies). After transfer to nitrocellulose membranes, the membranes were immunoblotted with the appropriate dilutions of the primary antibody: human α–syn (syn211, 1:1000; Santa Cruz Biotechnology, Santa Cruz, CA) and α–tubulin (DM1A, 1:2000; Santa Cruz Biotechnology, Santa Cruz, CA. Signals were visualized with fluorescent secondary antibodies and quantified with ImageJ.

### Statistical analysis

Statistical analysis was performed using Graphpad Prism 5 (Graphpad Software Inc. La Jolla, CA). Data were presented as mean ± SEM. Statistical significances were determined by comparing means of different groups and conditions using unpaired Student t–test or one–way ANOVA with post hoc Tukey test.

## Results

### Transgenic hα–syn is unable to undergo long–range propagation

**Fig. 1:**
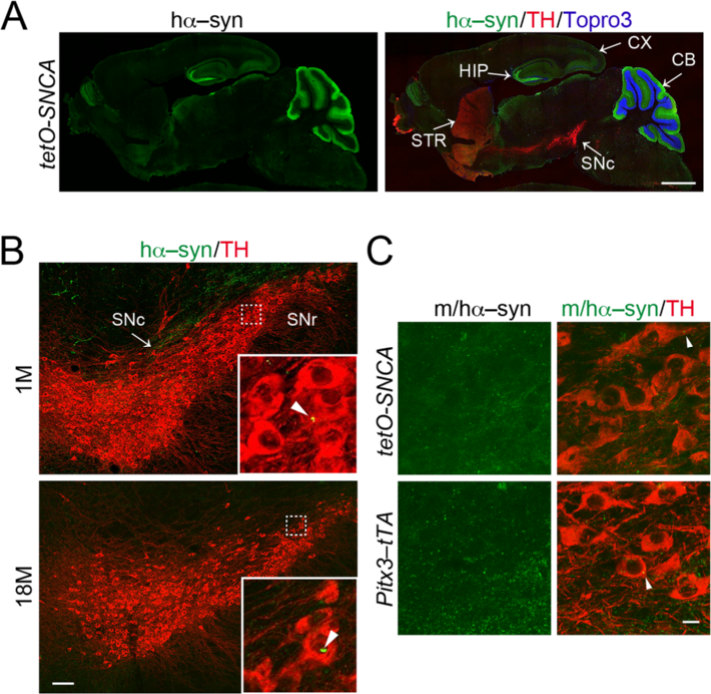
No propagation of α-syn into the nigrostriatal DA neurons of tetO–*SNCA* single transgenic mice. **a** Sample images show the expression pattern of hα-syn (green) in the sagittal sections of 1–month–old *tetO*–*SNCA* mice. DA neurons were marked by TH staining (red). Topro3 was used for counter–staining (blue). CX; cerebral cortex; HIP: hippocampus; CB: cerebellum; STR: striatum; SNc: *substantia nigra pars compacta.* Scale bar: 1 mm. **b** Sample images show the staining of hα-syn (green) and TH (red) in the SNc of 1– and 18–month–old *tetO–SNCA* mice. Insets highlight the boxed area. Arrowheads point to the hα-syn–positive puncta. SNr: *substantia nigra pars reticulata.* Scale bar: 100 μm. **c** Sample images show the staining of m/hα-syn (green) and TH (red) in the SNc of 18–month–old *tetO–SNCA* single transgenic and *Pitx3–tTA* heterozygous knock-in mice. Arrowheads point to the hα-syn–positive puncta. Scale bar: 10 μm

We generated a new line of human wild–type α–syn (*SNCA*) transgenic mice under the control of tetracycline operator (*tetO*): *tetO–SNCA*. The expression of transgenic *SNCA* assumes to be regulated by the *tetracycline transactivator* (*tTA*) in a “tet–off” gene expression system [17]. However, immunostaining revealed substantial expression of hα–syn in multiple brain regions, including the hippocampus, cerebellum, and cortex, independent of *tTA* (Fig. 1a). Our first test for propagation utilized the “leaky” expression of hα–syn present in *tetO–SNCA* transgenic mice. We wanted to see whether the tyrosine hydroxylase (TH)–positive SNc DA neurons that were initially devoid of hα–syn (Fig. 1a) would show any hα–syn accumulation with age. Potentially, these other tissues or residual hα–syn from the cerebrospinal fluid can aid in “infecting” the SNc DA neurons. If propagation can occur in this long–range fashion, hα–syn might accumulate in SNc DA neurons at advanced ages. We checked for this expression at 1 and 18 months of age (Fig. 1b). At 1–month–old, we found a few hα–syn–positive puncta distributed in the SNc region; some were spotted inside of SNc DA neurons (Fig. 1b, inset). Any additional hα–syn present at later ages would have been evidence for propagation. However, at 18 months of age, we were unable to see any apparent propagation (Fig. 1b). The lack of any substantial accumulation of hα–syn in the SNc DA neurons indicates that no long–range propagation is evident for *tetO*–*SNCA* mice.

Since the transcription factor paired–like homeodomain 3 (*Pitx3*) is only expressed by subpopulations of midbrain DA neurons [18], previously we inserted tetracycline transactivator (*tTA*) coding sequence into the 3’–untranslated region of mouse *Pitx3* gene to generate *Pitx3–tTA* knock–in mice, allowing *tTA* selectively expressed in midbrain DA neurons [16]. In this so–called “tet–off” system, tTA can turn on the expression of any transgene under the control of tetracycline operator (*tetO*) [16]. In the absence of such a transgene, this line of mice has no transgenic expression in the midbrain (Fig. 1a, b). Thus, to test whether hα–syn would induce the aggregation of endogenous mouse α–syn (mα–syn) in SNc DA neurons, we stained the midbrain sections of 18–month–old *tetO–SNCA* single transgenic and *Pitx3–tTA* heterozygous knock–in mice with an antibody that recognizes both mouse and human α–syn (m/hα–syn). We observed a similar number of small m/hα–syn–positive puncta in the SNc DA neurons of *tetO–SNCA* and control *Pitx3–tTA* mice (Fig. 1c), indicating a lack of recruitment of endogenous α–syn. Together, these observations suggest a lack of long–range transneuronal propagation of transgenic hα–syn into SNc DA neurons during aging.

### α–synuclein is not transmitted from presynaptic terminals into SNc DA neurons

**Fig. 2:**
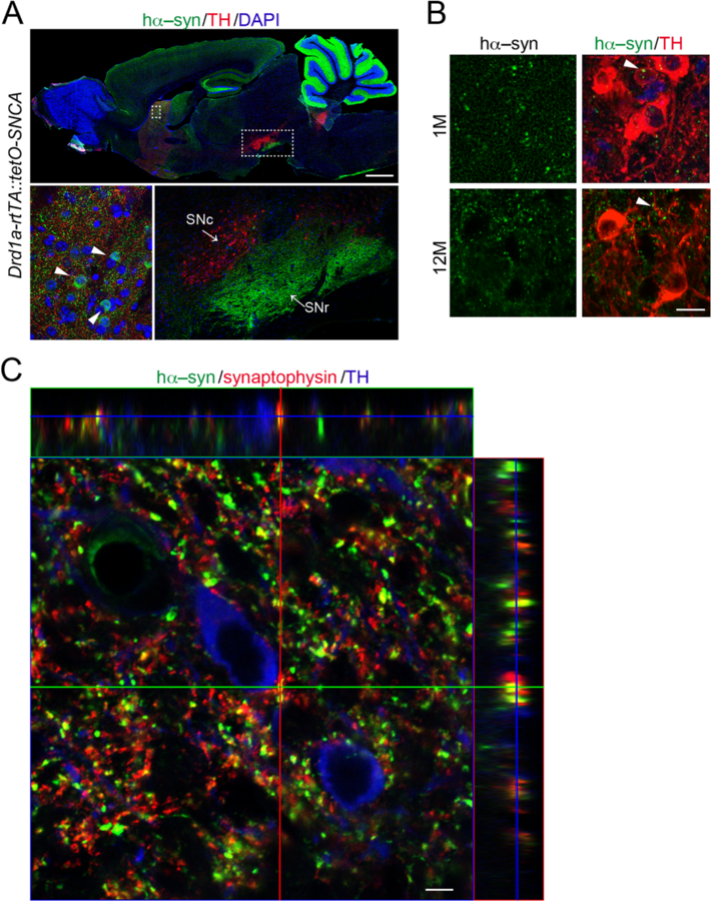
α-synuclein is not transmitted in anatomically connected regions. **a** In the top panel, sample image shows the expression pattern of hα-syn (green) and TH (red) in the sagittal sections of 1–month–old *Drd1a–rtTA::tetO*–*SNCA* bigenic mice. Topro3 was used for counter–staining (blue). In the bottom left panel, arrowheads point to Drd1–type striatal neurons that express hα-syn. The bottom right panel highlights the boxed area in the top panel. Scale bar: 1 mm. **b** Sample images show the staining of hα-syn (green) and TH (red) in the SNc of 1– and 18–month–old *Drd1a–rtTA::tetO*–*SNCA* bigenic mice. Arrowheads point to the hα-syn–positive puncta. Scale bar: 10 μm. **c** Sample image shows the staining of hα-syn (green), synaptophysin (red) and TH (blue) in the SNc of 18–month–old *Drd1a–rtTA::tetO*–*SNCA* bigenic transgenic mice. The panels at the top and right depict the distribution of different fluorophores along the Y– and X–axis. Scale bar: 10 μm

We next examined the transmission between anatomically connected brain regions, specifically the SNc and striatum in the basal ganglia. The majority of DA neurons in the SNc send projections to SPNs in the striatum [19]. SPNs comprise two main subpopulations that form direct and indirect pathways in the basal ganglia [19]. In the direct pathway, most SPNs that express dopamine receptor D1 (*Drd1*) send projections to neurons at *substantia nigra pars reticulata* (SNr), while some directly form synapses with SNc DA neurons [20, 21]. To further investigate propagation in the direct pathway, we used a line of mice that utilizes a *reverse tetracycline transactivator* (*rtTA*) and the *Drd1a* promoter, which directs transgene expression in the SPNs of direct pathway. When crossed with *tetO–SNCA* mice, we expect to see hα–syn expression along the direct pathway. Indeed at 1-month-old, *Drd1–rtTA/tetO–SNCA* mice showed strong hα–syn expression in the striatum and SNr, but no expression in the SNc (Fig. 2a). Once again, hα–syn expression at later ages would indicate that propagation is present. We then looked at 12–month-old mice and found that they too had no hα–syn–positive cells present in the SNc (Fig. 2b). As seen with the *tetO–SNCA* mice (Fig. 1b and c), small hα–syn–positive puncta were observed near or on top of SNc DA neurons (Fig. 2b). These puncta were also positive for synaptophysin, a marker for presynaptic terminals [22] (Fig. 2c), indicating a presynaptic enrichment of α–syn as previously documented [23]. The same as the previous experiments, we again found no indication that hα–syn possesses the ability to propagate across the synapses.

### α–synuclein is unable to undergo cell–to–cell transmission between SNc DA neurons

**Fig. 3:**
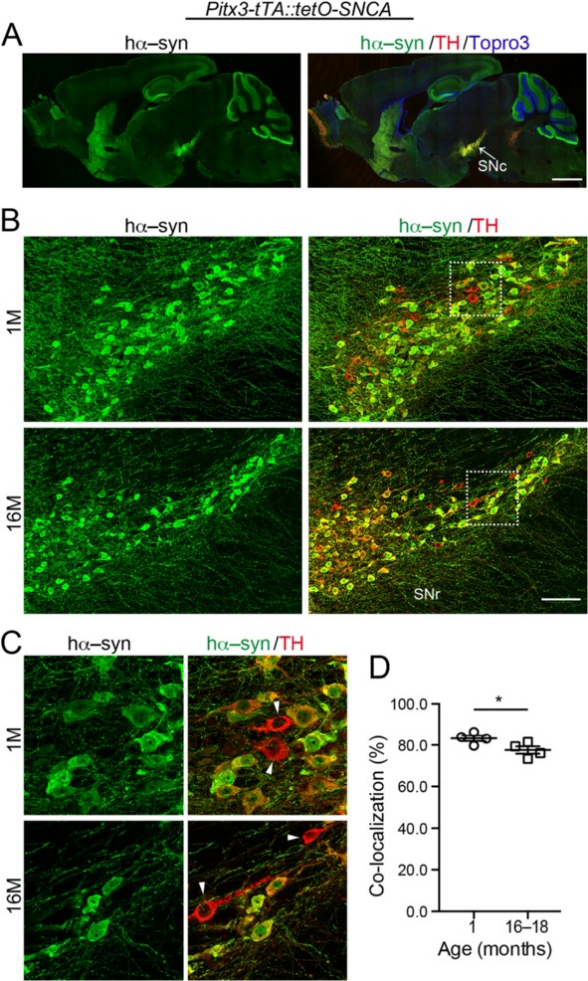
α-syn is unable to undergo cell–to–cell transmission at SNc. **a** Sample images show the expression pattern of hα-syn (green) and TH (red) in the sagittal sections of 1–month–old *Pitx3–tTA::tetO*–*SNCA* bigenic mice. Topro3 was used for counter–staining (blue). Scale bar: 1 mm. **b** Sample images show the staining of hα-syn (green) and TH (red) in the SNc of 1– and 16–month–old *Pitx3–tTA::tetO*–*SNCA* bigenic mice. Scale bar: 100 μm. **c** Images highlight the boxed areas in **b**. Arrowheads point to the hα-syn–negative DA neurons. **d** Scatter plot depicts co-localization percentages at 1 month and 16 months. Data were presented as mean ± SEM. *P < 0.05

We finally examined α–syn propagation within SNc DA neurons. To express *SNCA* in the midbrain, we crossbred *Pitx3–tTA* heterozygous knock–in mice with *tetO–SNCA* heterozygous transgenic mice to generate *Pitx3–tTA*::*tetO–SNCA* bigenic mice. The cells expressing hα–syn co–localize well with TH in the SNc in these mice (Fig. 3a). We subsequently examined co–localization patterns in the *Pitx–tTA*::*tetO–SNCA* bigenic mice that expressed hα–syn in midbrain DA neurons and (Fig. 3b-d). We looked at mice that were 1– and 16–18–month–old to determine how co–localization values changed with age. *Pitx3* is mainly expressed in the ventral SNc DA neurons, but not in the dorsal ones that account for about 20 % of total DA neuron population [24]. In our study, this translated to ~80 % of the TH–positive SNc cells expressing hα–syn under the control of the *Pitx3* promoter (Fig. 3d). We wanted to see if this percentage would increase with age, indicating the presence of cell–to–cell transmission of hα–syn in these cells. Contrary to what would be expected for propagation, we found that the average percentage of co–localized cells at 1–month–old was 83.3 %, whereas the percentage at 16–18–month–old was 77.6 % (Fig. 3d). As we did not see the increase that indicates the presence of propagation, this experiment provided no evidence for local propagation between neighboring SNc DA neurons.

**Fig. 4:**
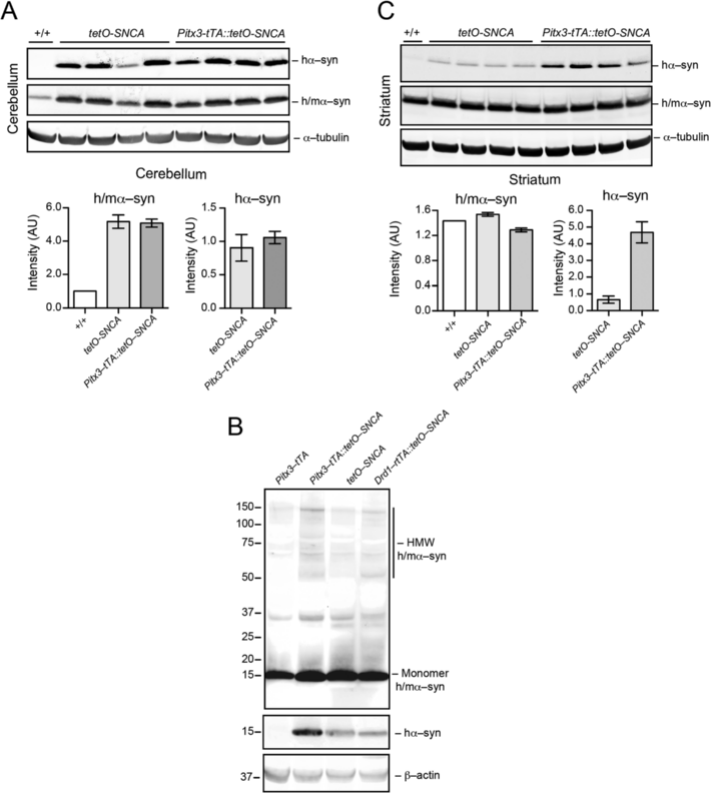
Overexpression of hα-syn in *tetO-SNCA* single and *Pitx3–tTA::tetO–SNCA* bigenic mice. **a** Western blots show expression of hα-syn and m/hα-syn in the cerebellum of 1–month–old *tetO-SNCA* single and *Pitx3–tTA::tetO–SNCA* bigenic mice. α-tubulin was used as loading control. Bar graphs depict the signal intensity. Data were presented as mean ± SEM. **b** Western blot shows expression of hα-syn and m/hα-syn in the whole brain of 18–month–old *Pitx3–tTA, tetO-SNCA* single, *Drd1a–rtTA::tetO–SNCA* bigenic and *Pitx3–tTA::tetO–SNCA* bigenic mice. β-actin was used as loading control. **c** Western blots show expression of hα-syn and m/hα-syn in the striatum of 1–month–old *tetO-SNCA* single and *Pitx3–tTA::tetO–SNCA* bigenic mice. α-tubulin was used as loading control. Bar graphs depict the signal intensity. Data were presented as mean ± SEM

In addition to tissue staining, Western blotting revealed more than 5–fold increase of α–syn expression in cerebellum of *tetO-SNCA* single and *Pitx3–tTA::tetO–SNCA* double transgenic mice (Fig. 4a). Furthermore, α–syn–positive high molecular weight (HMW) bands were also detected in the whole brain homogenates of *tetO-SNCA* single, *Pitx3–tTA::tetO–SNCA,* and *Drd1–rtTA::tetO-SNCA* double transgenic mice (Fig. 4b), suggesting the existence of α–syn aggregates in the mouse brains of all the transgenic lines. Western bolting also showed the hα–syn expression was substantially increased in the striatum of *Pitx3–tTA::tetO–SNCA* double transgenic mice compared to the *tetO-SNCA* single animals (Fig. 4c), resulting from the projection of hα–syn–expressing DA axons at the striatum (Fig. 3a).

## Discussion

We show here that transgenic hα–syn does not show detectable propagation to nigrostriatal DA neurons in various mouse models. We first used tetO–*SNCA* single transgenic mice to show that we could not observe long–range propagation of hα–syn into SNc DA neurons. These mice have no transgenic hα–syn expression in the nigrostriatal pathway; however, they do have “leaky”, non–specific hα–syn expression in other brain regions (i.e. hippocampus, cortex, and cerebellum). Any of these other regions could have played a role being a source of α–syn if the protein could indeed propagate. We performed immunohistochemical experiments on young and aged mice to see if we can observe hα–syn–positive staining anywhere in the SNc DA neurons of aged animals. However, these experiments gave no indication that long–range propagation was present in *SNCA* mice.

The following experiment tested propagation that may occur through neuronal projections from neighboring brain regions. For these experiments, we utilized a line of mice, *Drd1a–rtTA*::*tetO–SNCA*, which had hα–syn expression in the striatum and SNr, modeling the direct pathway of the basal ganglia. At 1–month of age, these mice exhibited no hα–syn expression in the SNc DA neurons. If propagation was present, we should be able to see hα–syn expression at later ages in these mice. This may have occurred as transmission directly to the SNc from the SPNs that form synapses onto SNc DA neurons [21]. However, as with the previous experiments, we found no evidence of hα–syn being present in the SNc, again showing that there was no evident propagation.

**Fig. 5:**
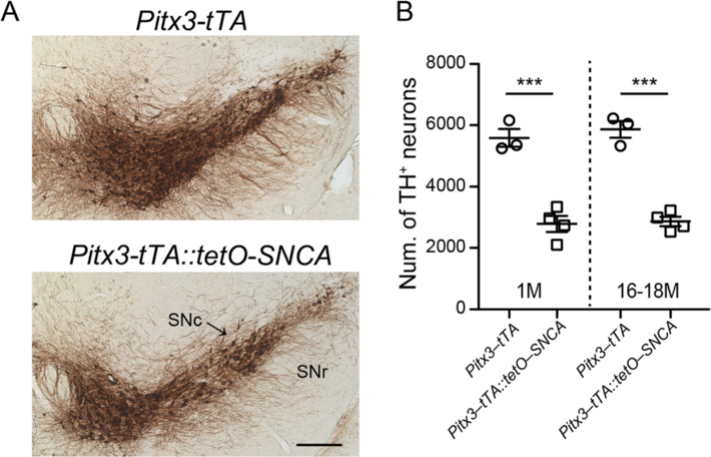
Loss of DA neurons in the SNc of *Pitx3–tTA::tetO–SNCA* bigenic mice. **a** Sample images show the staining of TH (brown) in the SNc of 16–month–old *Pitx3–tTA* heterozygous knock-in and *Pitx3–tTA::tetO*–*SNCA* bigenic mice. Scale bar: 100 μm. **b** Scatter plot depicts the number of remaining TH–positive neurons in the SNc of 1 month and 16–18 months. Data were presented as mean ± SEM. ***P < 0.001

Finally, we looked at *Pitx3–tTA*::*tetO–SNCA* bigenic mice, which utilize the *Pitx3* driver to promote hα–syn expression along the nigrostriatal pathway, in addition to the leaky expression patterns seen in the *tetO–SNCA* single transgenic mice. Following the expression pattern of *Pitx3*, we found that ~80 % of cells were both hα–syn–positive and TH–positive in 1–month–old bigenic mice. An increase in this percentage in aged mice would indicate that more cells were becoming hα–syn–positive, thus giving evidence for cell–cell transmission of α–syn. While there was about 50 % loss of SNc DA neurons in *Pitx3–tTA*::*tetO–SNCA* bigenic mice compared to *Pitx3–tTA* knock-in mice, no further degeneration occurred between 1–month and 16–18–month–old bigenic mice (Fig. 5a–b). Thus any increase could be attributed to the spread of α–syn, as opposed to cell death that may have resulted from α–syn toxicity. Instead of seeing the increase that would indicate propagation, we actually saw a slight decrease. However, the lack of degeneration led us to conclude that this decrease likely has no actual significance in the pathogenesis.

## Conclusion

Many studies have shown the ability of α–syn to propagate with the use of stereotaxic injections of preformed fibrils and have provided very convincing data for the ability of α–syn to behave as a prion, both in neurons and in glial cells [14]. However, these studies often take advantage of artificial injections or inoculations, which may not be as applicable in a clinical, physiological setting. Therefore, alternative explanations to the prion hypothesis cannot be dismissed, including oxidative stress, excitotoxicity, neuroinflammation, and loss of neurotrophic factor support. These alternative explanations are not mutually exclusive and may potentially induce pathogenesis in a synergistic manner. Future studies should focus on microglial activation and other inflammatory responses in the brain resulting from intracerebral injections and inoculations. In addition, further scrutiny into the effect of inflammation on α–syn expression can provide answers about the causes and mechanisms by which α–syn adopts its abnormal prion–like qualities.
